# Towards a socially just model: balancing hunger and response to the COVID-19 pandemic in Bangladesh

**DOI:** 10.1136/bmjgh-2020-002715

**Published:** 2020-06-01

**Authors:** Sabina Faiz Rashid, Sally Theobald, Kim Ozano

**Affiliations:** 1 BRAC University James P Grant School of Public Health, Dhaka, Bangladesh; 2 International Public Health, Liverpool School of Tropical Medicine, Liverpool, UK; 3 Liverpool School of Tropical Medicine, Liverpool, UK

**Keywords:** public health, prevention strategies, health services research, health policy

**Summary box**

- Responsive and timely research is needed to better understand the challenges faced by poor and vulnerable populations to inform immediate interventions and policies to address this unprecedented COVID-19 modern-day pandemic.
- There is a need to research changes through time to understand and address the continuous and long-term economic, mental and emotional impact of lockdown on the most marginalised.
- Many of the Bangladeshi population are vulnerable, yet the COVID-19 response focuses on individual behaviour with limited attention to the social, economic and contextual factors that prevent the most marginalised from following national recommendations.
- In the context of structural constraints, continuation of the lockdown has to be accompanied by strong political resolve to ensure that people do not go without basic meals and have basic health information and support.
- The experiences of people living and working in slums in Bangladesh needs to be captured and translated to context specific strategies for lockdown, as current measures risk starvation for many.
- In the context of COVID-19, the lockdown model is being imported from a different context (western or developed economies) with stronger economic bases and better social safety nets for those in need, but is there a better way forward for low resource contexts?
- Economic mortalities may overtake health mortalities for the poorest who survive on daily wage labour.

## Rapid responsive research in Bangladesh is revealing the realities of lockdown for the poor and vulnerable

In Bangladesh, the James P. Grant School of Public Health is undertaking responsive research to try and understand the needs of the population during COVID-19. The multidisciplinary research includes 80 case studies in urban slums to capture the lived experiences and the impact of shutdown of the people living and working in Dhaka during COVID-19. In addition, a rapid large scale urban/rural survey is being conducted via phone interviews, with follow-ups, aimed to assess the possible effects of the pandemic on several domains of a household or family such as consumption, income, health, coping strategies, psychological well-being and gender. The survey takes a dynamic approach: questions are modified based on current understandings and relevant emerging issues related to the crisis. With a focus on marginality, interviews have taken place with the transgender group of people commonly known as ‘Hijra’ in South Asian countries and with street workers including adolescence and young adults.

Reading these data alongside media reports and articles on the coronavirus pandemic, one is overcome with a range of emotions: depression, paralysis, anger, denial and helplessness; emotions that are reflective of being privileged and of having the luxury to dwell on them. For the vast numbers of the poor, microbusiness owners, labourers, transport workers, informal sector employees and many other groups who depend on daily wages/earnings and have no social safety net, there is now only the pain of hunger, not figuratively, but literally. With the shutdown now extended to a month, these groups are under real threat of starvation. There are international conventions and declarations on the right to food, on the right to be free from hunger.^1^ Yet the world suffers from an estimated 9 million people dying of hunger and hunger related diseases annually, more than AIDS, malaria and tuberculosis combined.^2^ It is the world’s biggest health problem, and with entire countries and economies now under lockdown, it risks getting much worse for those who live in difficult environments. Although Bangladesh has achieved a lot over recent decades, with improved availability of food due to increased production, 40 million people—one quarter of the population—remain food insecure, and 11 million suffer from acute hunger.^3^ These figures will worsen after the impact of COVID-19.

## For many, every day is a battle: COVID-19 is one addition to a long list of challenges for survival

**Figure 1:**
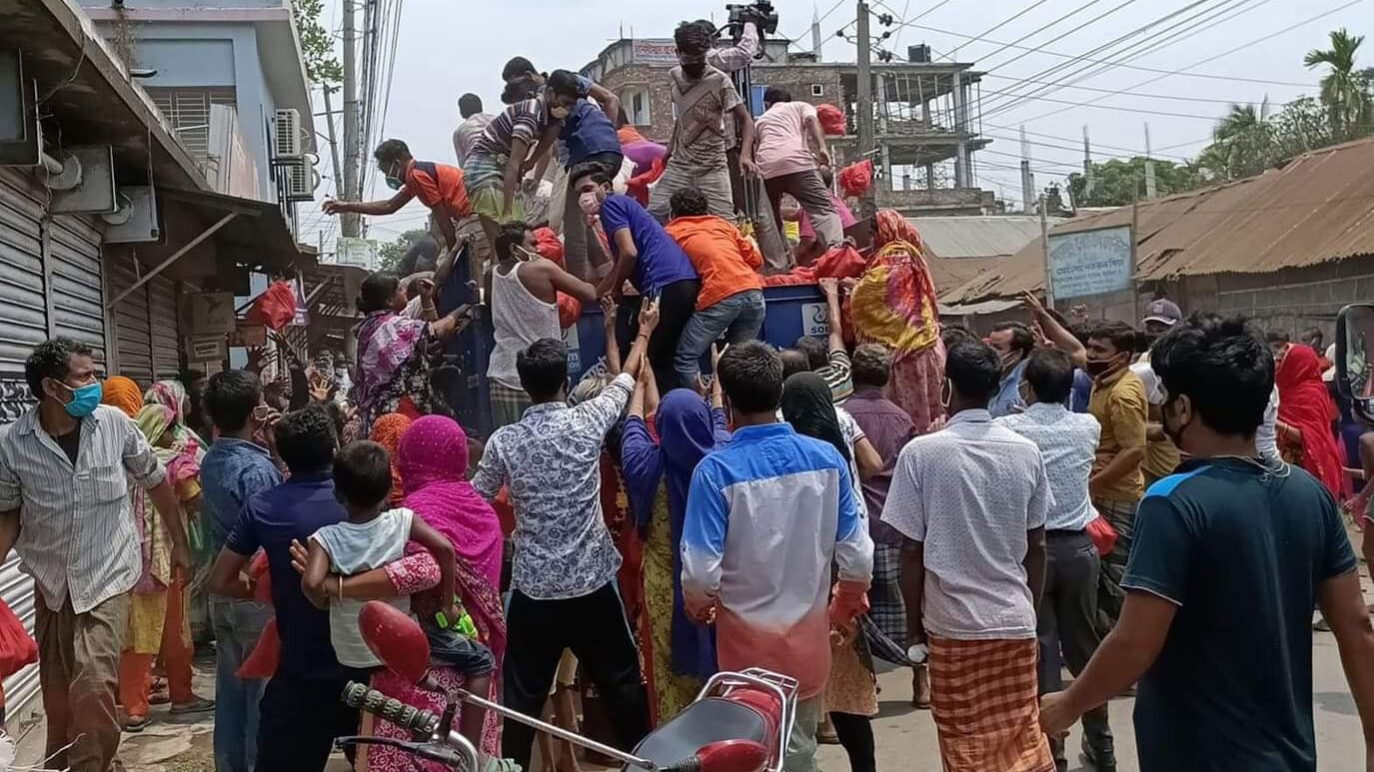
Looting for Food: Desperation Sets In.

The poor and the vulnerable with their erratic and meagre earnings somehow manage to keep fighting and living and demonstrating impressive resilience, being confronted with illnesses and deaths is an everyday reality for many. While there is fear of the coronavirus, there is also the acceptance that it is yet another addition to an already long list of health challenges that they face. Furthermore, with access to their sparse resources being severely constrained or denied as a result of the shutdown, for many, the immediate threat to consumption for survival, and not necessarily the pandemic, is becoming a greater concern. BRAC’s (NGO) conducted a rapid perception survey on COVID-19 conducted between 31 March 31 and 5 April 2020 for instance found that 18% and 10% of urban and rural respondents, respectively, had no food stored at home, while 37% and 21%, respectively, had only 1–3 days food reserve.^4^ Figure 1 shows ‘looting goods from a truck carrying relief’ *(source: photo TBS (The Business Standard), 12 April 2020*).

## The focus on individual behaviour prevents the poorest from following national recommendations

Health bodies and various governments have been promoting different measures to contain the pandemic that focus on individual behaviour with little attention to the social, economic and contextual factors. Public health preventions tend to be based on the biomedical virus and individual determinants of health, whereas for millions, the stark living conditions, social and contextual inequalities and realities of how and where they live prevent them from following such recommended guidelines. There needs to be a recognition of the complexity of factors that underlie and impact on marginalised populations lives. Practising social distancing, washing of hands with soap and staying at home are all very well for the privileged who can afford to do so; however, for the poor with 5–6 or more members crammed in one room within slums, sharing irregular water supply, communal latrines and cooking spaces, in some of the dirtiest and densest places on earth, such messaging needs urgent adaptation to reflect the realities of context and support is critical.

The poor and vulnerable already live on the edge. The added stress of the pandemic combined with prolonged shutdowns will amplify further their despair and hopelessness. While health is a very real concern, for Bangladesh to sustain the shutdown requires all of us to focus all of the country’s resources on ensuring that no one goes without food. We have to believe the rest will follow, once this is ensured. If not, as Nobel Laureates Esther Duflo and Abhijit Banerjee highlighted with respect to the situation in India, the poor and the vulnerable will be left with no choice but to break the shutdown for their livelihood.^5^

The last interview of an adolescent street peddler stated, ‘*how much longer? We heard four more days. We have no food, no money*’. These narratives are typical for most of the poor families we interviewed, in similar distress and concerns were echoed, much more in the urban surveys compared with the rural surveys (for now) and case studies in Dhaka city urban settlements with mainly the informal workers, who are dependent on daily wages to survive. Try imagining, if you can, the gut-wrenching panic and anxiety, when many of them learn it will be an additional 10 days or more. Rumours that the shutdown may continue until end of April or even May is going to lead to unimaginable consequences on the poorest and for the country as a whole. We need a socially just model to tackle this pandemic, and this requires us to acknowledge the fault lines that exist in our underlying assumptions as well as the very real inequities that exist between the poorest and others.

## Political commitment for economic support for the poor needs urgent and effective implementation

Bangladesh, like many other countries, has rolled out an economic stimulus package to address the severe economic and business fallout from the pandemic. The government is also in the process of unveiling support for the poor. This scheme will also include support for farmers who are critical for ensuring the food supply chain for all of us—the rich, the middle class and the poor. While this package should really have been the first step taken by the state, it now needs to be implemented efficiently, systematically and equitably. There are numerous articles and reports detailing the mismanagement,^6 7^ favoured groups in communities and a complete lack of coordination between different bodies involved in distributing the initial state funded food and/or cash aid programmes.^8–10^ This has to stop. While there is no easy solution or strategy, for Bangladesh and its high proportion of vulnerable populations, continuation of the shutdown has to be accompanied with strong political resolve to ensure that people do not go without food and have basic health information and support, given the grounded realities of their lives. Otherwise, it will be the final nail in the coffin for the poor and maybe even beyond. The trauma and enormity of what will unfold if this is not done properly cannot be emphasised enough.

The shutdown or lockdown model has been imported from western or developed economies with stronger economic bases and better social safety nets for those in need. But is it the only way forward? China, Hong Kong, Singapore, countries that were successful in containing the first wave, are now facing a resurgence largely due to infections coming from outside travellers, and some countries have begun reinstating containment measures again.^11^ How long can a shutdown be sustained in a largely different context? While this is an entirely unknown territory, Iran’s president for instance declared that ‘low-risk’ economic activities will resume from April 11 in spite of the virus not being contained.^12^ The Iranian government is thus balancing the risks of the pandemic versus further wrecking a sanctions battered economy. Sadly, countries with large pools of poor populations may soon be forced to confront similar trade-offs, with all its moral and ethical implications, if there is no solution soon in sight.

The political and social actions taken now at the global, national, subnational and local levels to understand and meet the needs of the urban poor are essential to addressing the current pandemic and also in preventing a post-COVID-19 rise in people experiencing extreme poverty and death from the wider social determinants of health. If action is taken now, there is a chance to learn and build cities that are more resilient and responsive to future crises. Having a responsive research agenda is the first step to informing, developing and delivering policies and strategies that are informed by data, within Lower Middle Income Countries (LMICs) and in all countries and contexts where inequities exist. However, these must be developed in partnership with civil society organisations, community leaders/gatekeepers and residents who know what is needed to make a difference, now and in the future. There is also a need to engage in cross-country discussions to share learnings from previous emergency responses in urban settings and support sharing and solidarity around current promising strategies across and between different contexts.
